# Metal-polyphenol networks-modified tantalum plate for craniomaxillofacial reconstruction

**DOI:** 10.1038/s41598-024-51640-4

**Published:** 2024-01-10

**Authors:** Zhengyu Wei, Zhisen Shen, Hongxia Deng, Tairong Kuang, Jinggang Wang, Zhipeng Gu

**Affiliations:** 1 Department of Otorhinolaryngology Head and Neck Surgery, the Affiliated Lihuili Hospital, Ningbo University, Ningbo, 315040 Zhejiang China; 2 Department of Otorhinolaryngology Head and Neck Surgery, Ningbo Medical Centre Lihuili Hospital, Ningbo, 315040 Zhejiang China; 3 Health Science Center, Ningbo University, Ningbo, Zhejiang China; 4 College of Material Science and Engineering, Zhejiang University of Technology, Hangzhou, Zhejiang China; 5 Laboratory of Bio-Based Polymeric Materials Technology and Application of Zhejiang Province, Ningbo Institute of Materials Technology and Engineering, Chinese Academy of Sciences, Ningbo, 315201 Zhejiang China; 6 College of Polymer Science and Engineering, State Key Laboratory of Polymer Materials Engineering, Sichuan University, Chengdu, Sichuan China

**Keywords:** Biomedical engineering, Biomaterials

## Abstract

Using three-dimensional (3D) printing technology to make the porous tantalum plate and modify its surface. The physicochemical properties, cytocompatibility, antioxidant capacity, and histocompatibility of the modified materials were evaluated to prepare for the repair of craniomaxillofacial bone defects. The porous tantalum plates were 3D printed by selective laser melting technology. Tantalum plates were surface modified with a metal polyphenol network. The surface-modified plates were analyzed for cytocompatibility using thiazolyl blue tetrazolium bromide and live/dead cell staining. The antioxidant capacity of the surface-modified plates was assessed by measuring the levels of intracellular reactive oxygen species, reduced glutathione, superoxide dismutase, and malondialdehyde. The histocompatibility of the plates was evaluated by animal experiments. The results obtained that the tantalum plates with uniform small pores exhibited a high mechanical strength. The surface-modified plates had much better hydrophilicity. In vitro cell experiments showed that the surface-modified plates had higher cytocompatibility and antioxidant capacity than blank tantalum plates. Through subcutaneous implantation in rabbits, the surface-modified plates demonstrated good histocompatibility. Hence, surface-modified tantalum plates had the potential to be used as an implant material for the treatment of craniomaxillofacial bone defects.

## Introduction

Craniomaxillofacial bone tissue defects can be caused by accidents, tumors, or congenital defects, leading to craniomaxillofacial deformities and severe functional impairments^1^. It also has a strong psychological impact on the patients^2^. The key to treatment is to repair the craniomaxillofacial bone tissue defects and restore the normal structure and function^3^. Craniomaxillofacial surgery implant materials, including medical metals, inorganic nonmetals, polymers, and composite materials, etc., have become an important part of clinical treatment. Tantalum metal has excellent corrosion resistance as well as high melting point, high strength, and wear resistance^4^. Recent studies have shown that tantalum is more biocompatible than titanium^5^. Traditional craniomaxillofacial metal implants are usually produced using manual techniques, which are not only laborious but also cause metal deformation and shape deviation during processing, resulting in poor therapeutic effects^6^. Three-dimensional (3D) printing is a rapid prototyping technique based on digital models, which creates 3D structures layer by layer^7^. Metal implants produced using 3D printing technology can be customized according to the patient's anatomical features^8^ and have a high matching accuracy^6^. However, metal implants currently used in craniomaxillofacial surgery are associated with a high risk of postoperative infection and insufficient long-term biological function. Improving the biocompatibility, anti-inflammatory ability, and binding force of craniomaxillofacial implants with living tissues is a scientific problem that requires attention. In recent years, the development of surface modification technology has aimed to improve the properties of metal biomaterials, especially their biocompatibility^5,9^, thereby expanding the range of applications of these metal biomaterials.

Bone defects are often accompanied by infection, and the treatment of infectious bone defects is still a clinical challenge because the blood supply in the infected area is limited and the self-healing ability of infectious bone defects is poor, resulting in a poor clinical prognosis^10–12^. Metal-polyphenol network (MPN) is a new organic–inorganic hybrid network system developed in recent years. MPN combines the specific functions of metal ions and polyphenol ligands, demonstrating distinctive benefits specifically designed to meet the desired characteristics of orthopedic biomaterials^10^. Natural polyphenols possess an abundance of phenolic hydroxyl groups, which confer tissue adhesive, antioxidant, and anti-inflammatory properties, alongside the ability to chelate metal ions^10,13,14^. Moreover, certain polyphenols exhibit inherent antimicrobial properties^10,15,16^, which is hypothesized to be linked to the precipitation of bacterial cell membrane proteins through the reaction of polyphenols^17^. Furthermore, metal ions contribute supplementary functions to MPN. Due to the coordination between polyphenol ligands and metal ions, MPN exhibits excellent anti-inflammatory, antioxidant, and antibacterial properties, and has been applied in the fields of skin repair, bone regeneration, and medical devices^5,10^. Lee et al. developed an MPN coating composed of epigallocatechin gallate and Mg^2+^ for surface modification of orthopedic titanium implants to enhance bone-implant interface bonding and improve osseointegration^18^. In particular, Zn^2+^ is a commonly used metal ion in MPN. Previous studies have shown that Zn^2+^ may play an antioxidant role by participating in the formation of the catalytic structure and function of superoxide dismutase (SOD), and a deficiency in Zn^2+^ is strongly correlated with heightened oxidative damage to lipids, proteins, and DNA^19^. Additionally, Zn^2+^ plays an anti-inflammatory role by regulating the activity of SOD, matrix metalloproteinases, and phosphodiesterase^19^. Zn^2+^ also has antibacterial, immune, cell metabolism, and osteogenic properties, and is widely used in orthopedic materials^5,10,20,21^. Hence, it can be inferred that the presence of Zn^2+^ in MPN is likely to create advantageous circumstances conducive to the process of bone regeneration.

**Figure 1:**
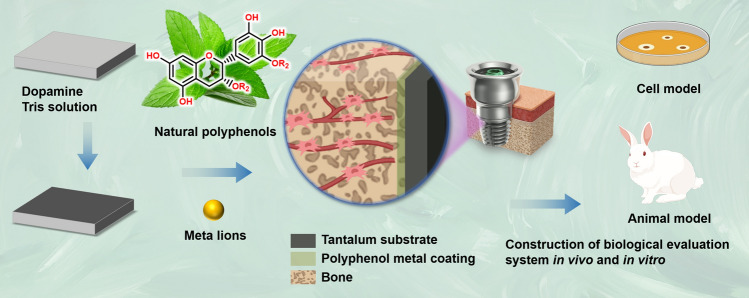
The graphical abstract of the study.

In this study, tantalum plates were modeled using 3D printing technology, and then the tantalum surface was modified by polyphenol coating and polyphenol zinc coatings. Next, the surface morphology, physicochemical properties, cytocompatibility, antioxidant capabilities, and histocompatibility were systematically investigated. Experiments have proved that the surface-modified tantalum plate material has excellent cytocompatibility, antioxidant capabilities, and histocompatibility, that can protect cells from oxidative damage, which is beneficial to craniomaxillofacial reconstruction. The graphical abstract of this study is shown in Fig. 1.

## Materials and methods

### Preparation and surface modification of the tantalum plates

**Figure 2:**
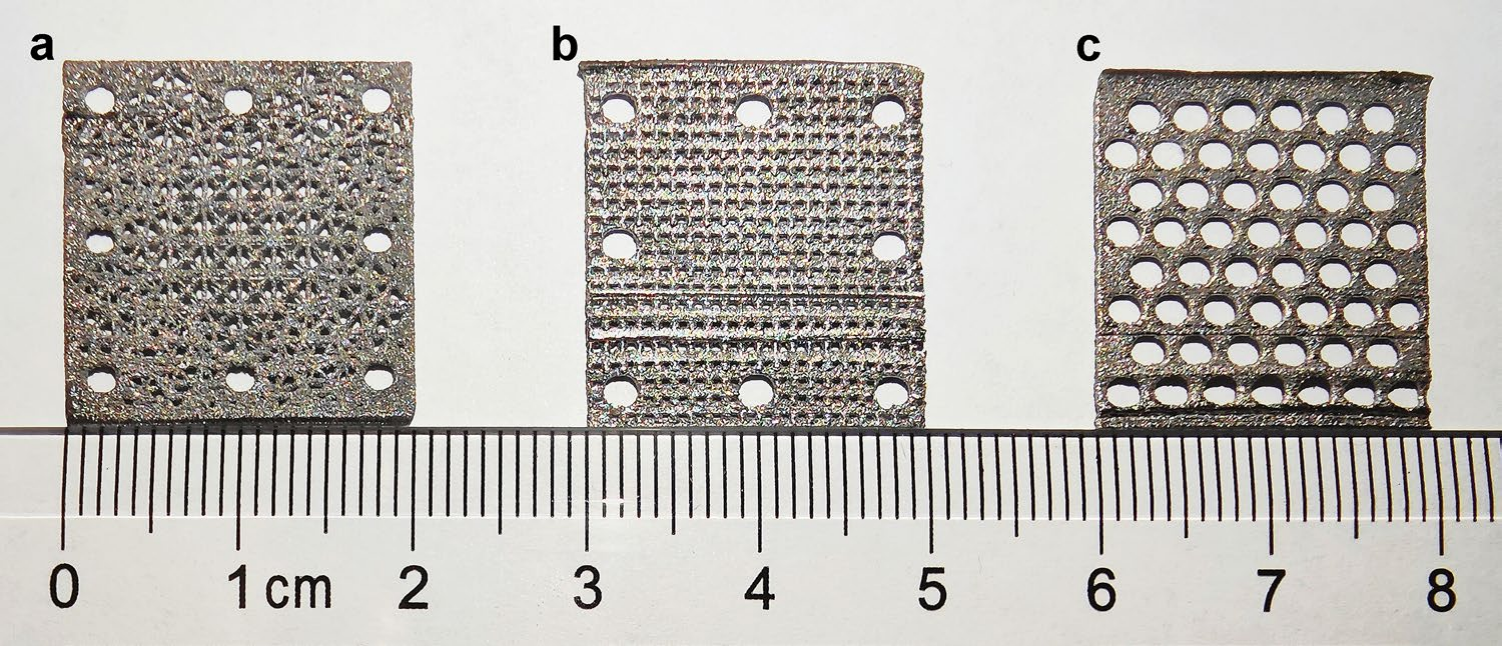
3D printed tantalum plates. (**a**) Tantalum plate with a random network structure. (**b**) Tantalum plate with uniform small pores. (**c**) Tantalum plate with uniform large pores.

The tantalum powder used in this study had a sphericity of 0.93, a bulk density of 10.05 g/cm^3^, and a Hall flow rate of 6.93 s/50 g. The selective laser melting equipment used was a laser rapid prototyping machine (EOS M290, Germany) with a total laser power of 400 W, a water-cooled laser, a spot diameter of 80 μm, a powder laying speed of 500 mm/s, a platform temperature of 100 ℃, and argon was used as a protective gas, with an inert gas consumption of ≈0.6 m^3^/h during processing. Three tantalum-plate models were designed. Figure 2a exhibits a randomly networked tantalum plate with dimensions of 20 mm × 20 mm × 0.3 mm, a mesh rib thickness of 0.3 mm, and a large pore for installation with a diameter of 2 mm. Figure 2b shows a tantalum plate with uniform small pores, with similar dimensions of 20 mm × 20 mm × 0.3 mm, but a small pore diameter of 0.5 mm, a pore spacing of 1 mm, and a large pore for installation with a diameter of 2 mm. Figure 2c shows a tantalum plate with uniform large pores, with dimensions of 20 mm × 20 mm × 0.3 mm and a pore diameter of 2 mm.

The MPN was chosen to modify the tantalum plates. Briefly, 50 mg of dopamine hydrochloride (Aladdin, USA) was dissolved in 10 ml of Tris–HCl buffer (10 mM, pH 8.5) and added to a tantalum plate. After 24 h, the tantalum plate was removed and thoroughly washed. Then, 200 mg of protocatechuic aldehyde was dissolved in 10 mL of water with 400 μL of ethanol, and 30 mg of zinc chloride was added. Afterwards, the coating for the plate was carried out in a shaking reactor at ambient temperature for 48 h, and finally a surface-modified tantalum plate was obtained^22–24^. No plants were used in this study.

### Mechanical performance testing

The compression test specimens consisted of tantalum plates with a random network structure, uniform small pores, and uniform large pores. Each group of tantalum plates (n = 6) had a standardized design size of 20 mm × 20 mm × 0.3 mm. The actual size was measured by using a high-precision electronic digital display caliper (Deli DL3944, China). The samples were subjected to vertical compression at room temperature with a preload of 300 N and a test speed of 0.5 mm/min using a Universal Materials Testing Machine (ZwickRoell, Germany). The load and compressive strength required to deform the specimen by 80% were measured.

### Morphological observation

The coating surface morphology was examined by using an FEI Quanta 250 scanning electron microscope (SEM) in backscattered electron mode (2.5 nm, 30 kV) with an acceleration voltage of 10 kV. The samples were washed three times with deionized water, dried with nitrogen flow, and sprayed with gold 24 h before the experiment.

### Surface hydrophilicity and hydrophobicity testing

Hydrophilic and hydrophobic of coatings were measured using Data Physics OCA 25 and recorded using DROP Image Advanced v 2.8 software at 25 °C. Deposit a 4 µL droplet of water on the substrate and the contact angle was measured within 5 s. All the test samples were applied with different coating substrates, washed 3 times with deionized water, and dried under a nitrogen flow before testing.

### Compatibility evaluation of the coated tantalum plate cells

Mouse embryonic fibroblasts (NIH-3T3) were cultured in high glucose Dulbecco's Modified Eagle's Medium supplemented with 10% fetal bovine serum and 1% penicillin–streptomycin. NIH-3T3 cells between the 3rd and 7th passages were used, and the culture medium was changed every two days. The interaction between the coated tantalum plate and the cells was assessed using live/dead cell staining. Cultured cells were seeded into 6-well plates at an appropriate density (20,000 cells per well) and incubated overnight until cells adhered to the wells. Then the extract solution of the tantalum plate (20 mg/mL) was added^25–27^. After 1 and 5 days of treatment and incubation, live/dead cell staining analysis was performed according to the manufacturer's protocol. For quantitative evaluation, cells were seeded into 96-well cell culture plates, incubated overnight, and then added to the extraction solution. After 1 and 5 days of incubation at 37 °C, the cell viability was measured using thiazolyl blue tetrazolium bromide (MTT), and absorbance was used as an indicator of cell survival.

### Measurement of intracellular reactive oxygen species (ROS) levels

2', 7'-Dichlorofluorescein diacetate (DCFH-DA) is a non-fluorescent compound that reacts with intracellular free radicals to generate the fluorescent product dichlorofluorescein. In this study, we monitored the production of ROS radicals and found that the fluorescence intensity depends on the amount of ROS radicals in the cellular environment. NIH-3T3 cells were seeded into 6-well plates and incubated overnight. Subsequently, the cell culture medium was removed, and the adherent cells were incubated with extract solution (20 mg/mL) and 9.8 µmol/L of H_2_O_2_ at 37 °C for 1 day and 5 days. Cells were then incubated with DCFH-DA for 30 min at 37 °C. After washing 3 times with PBS, intracellular ROS levels were monitored using an inverted fluorescence microscope.

### Detection of cellular oxidative stress indicators

#### *Determination of reduced glutathione (GSH)*

Total glutathione and oxidized glutathione (GSSG) were measured using a microcolorimetric assay kit (Beyotime Biotechnology Research Institute, China). The cells were washed 3 times with PBS, centrifuged, and the supernatant was aspirated. Three times the volumes of deproteinized M solution were added to the cells and thoroughly mixed. Samples were subjected to two rapid freeze–thaw cycles using liquid nitrogen and a 37 °C water bath. They were then placed in a 4 °C environment for 5 min. Then, the mixture was centrifuged at 10,000 × g for 10 min at 4 °C. Supernatants were collected for total glutathione measurement. The amount of GSSG was subtracted from the total glutathione to determine the amount of GSH.

#### Determination of the SOD activity

The SOD activity was determined using a WST-8 SOD detection kit (Beyotime Biotechnology Research Institute, China). Cells were collected by centrifugation at 1000 × g for 5 min, precooled at 4 °C, washed with physiological saline, and then disproportionated into sample preparation solution with superoxide. Cells were completely broken by pipetting. Then, they were centrifuged at 12,000 × g for 10 min at 4 °C, and the supernatant was collected as the test sample. For blank-1 (buffer + WST-8 working solution + initial solution), blank-2 (buffer + WST-8 working solution), and blank-3 (sample + buffer + WST-8 working solution) were also measured and the sample results were calculated according to the manufacturer's instructions. SOD activity was expressed as U/mg protein.

#### Determination of malondialdehyde (MDA)

The MDA level in NIH-3T3 cells was measured using the MDA assay kit (Beyotime Biotechnology Research Institute, China). Western and IP Cell Lysis Buffers were used to lyse cells. After cell lysis, samples were centrifuged at 12,000 × g for 10 min, and the supernatant was collected for subsequent measurements. The total protein level was determined using the BCA protein concentration assay kit, and the MDA level was expressed as nmol/mg of protein.

### Animal experiments

#### Animals

Nine male New Zealand rabbits, weighing 2–3 kg, were provided by the Jianfei Experimental Rabbit Breeding Farm in Simen Town, Yuyao City. The animals were fed ad libitum and had free access to water. Food and water were provided by the Experimental Animal Centre of Ningbo University. The rabbits were randomly divided into three groups (blank tantalum, polyphenol-coated, and polyphenol-zinc-coated groups; three rabbits per group). The animal experimental protocol was approved by the Ethics Committee of Ningbo University (approval number: 11576). All methods were carried out in accordance with relevant guidelines and regulations. All methods are reported in accordance with ARRIVE guidelines (https://arriveguidelines.org).

#### Materials and reagents

The materials used mainly include blank tantalum plates, polyphenol-coated tantalum plates, and polyphenol-zinc-coated tantalum plates. The main reagents used were 2% lidocaine hydrochloride, 2% iodophor, 0.9% normal saline, PBS buffer, and 10% neutral formalin.

#### Animal model establishment

Experimental animals were confined to the left-lateral position on the operating table. The fur around the right thigh was shaved, and the surgical area and surrounding skin were disinfected with 2% iodophor. Sterile towels were placed around the area and local infiltration anesthesia was induced using 2% lidocaine hydrochloride. A 3 cm incision was made, and a subcutaneous pocket was bluntly dissected under the fascia. A tantalum plate was implanted into the pocket, and the surgical incision was closed and disinfected with iodophor.

#### Postoperative treatment and observation

The experimental rabbits were housed and fed in cages after surgery. Four months after the surgery, a 3 cm incision was made at the original surgical site under local anesthesia, and whether the tantalum plate was still in place was observed and recorded. The implanted tantalum plate and surrounding tissues were removed, and the incision was closed and disinfected with 2% iodophor. The removed materials and tissues were washed with PBS prior to fixation with 10% neutral formalin. After surgery, the animals were euthanized by air embolism. After removing the tantalum plates, the tissues from the implantation site were embedded in paraffin. Hematoxylin and eosin (HE) staining was used to evaluate the inflammatory response of the observed tissue. Moreover, immunohistochemical staining was performed to further confirm the inflammatory response. The EnVision two-step method was employed for immunohistochemical staining of the paraffin-fixed samples. The samples were prepared through the incubation of tissue slices in EDTA antigen repair fluid at a temperature of 100 °C for 20 min, followed by subsequent cooling to room temperature. Antibodies for CD86 and TNF alpha were purchased from Abcam (Cambridge, UK) and used at a working concentration of 1:100. The CD80 antibody was purchased from Proteintech (Wuhan, China) and used at a working concentration of 1:200. The second antibody, DAB + chromogen and its substrate buffer were all purchased from Dako (Glostrup, Denmark). The sections were observed using a digital pathology slide scanner (Jiangfeng, China) at 400 × magnification.

### Statistical analysis

Statistical analyses were performed using the Origin version 2022 software, which can also be used for visualization.

## Results and discussion

### Influence of pore structure design on mechanical properties of tantalum plates

We designed and printed random network structure tantalum plates with 0.3 mm grid reinforcement, uniform small-pore tantalum plates with 0.5 mm pore diameter, and uniform large-pore tantalum plates with 2 mm pore diameter. The mechanical properties of materials used to manufacture maxillofacial prostheses affect the outcome of restorative treatments^28^. Previous studies have shown that the mechanical properties of materials are affected by their pore size^29^. Here, we compared the load and compression strength of tantalum plates with three different pore structure designs.

Table 1 shows the load and compressive strength at 80% deformation of tantalum plates with three different pore structure designs. The tantalum plate with a random network design was carrying a load of 7.24 ± 1.23 kN and had a compressive strength of 16.56 ± 2.82 MPa at 80% deformation, while the tantalum plates with uniform small and large pores were carrying loads of 9.29 ± 0.82 kN and 4.79 ± 0.43 kN and had compressive strengths of 21.94 ± 1.81 MPa and 11.59 ± 0.79 MPa, respectively. Therefore, different pore structure designs affect the mechanical properties of tantalum plates.

**Table 1:** The mechanical testing of tantalum plates.

Pore structure design	Maximum load F max (kN)	Compressive strength (MPa)
Random network	7.24 ± 1.23	16.56 ± 2.82
Uniform small pores	9.29 ± 0.82	21.94 ± 1.81
Uniform large pores	4.79 ± 0.43	11.59 ± 0.79

Uniform small-pore tantalum plates have the highest compression strength, while uniform large-pore tantalum plates have the lowest compression strength. The tantalum plates with uniform small pores have higher compressive strength than those with the other two pore structure configurations, indicating that plates with uniform small pores may be the optimal choice for reconstructing the mandible and other parts that must withstand gravity. Moreover, although porous polyethylene has been used to treat orbital floor fractures, the risk of infection and migration is high. Our tantalum plates were designed with a network structure that could promote soft tissue integration and reduce the risk of migration^30^, making it an excellent repair material for orbital floor fractures.

### Surface properties of the coated tantalum plates

However, many biomaterials have low biocompatibility with host tissues and may cause inflammatory and immune responses or foreign body reactions after implantation^31^. Surface modification techniques can improve the cytocompatibility of biomaterials. In this study, we used selective laser melting technology to print tantalum plates with different network structures. It has been reported that polydopamine can be produced by self-polymerizing dopamine on the surfaces of almost all solid materials and serve as a versatile secondary reaction platform, which is widely used in surface modification of biomaterials^32^. Moreover, the metal-binding ability of catechols in polydopamine could contribute to the reduction of metal ions^33^. After treatment of tantalum plates with dopamine, we creatively introduced polyphenol coatings and polyphenol zinc coatings to modify their surfaces to improve their cell compatibility. A new implant scheme was proposed for cranial and jaw bone repair and reconstruction. The biocompatibility of a biomaterial is affected by its surface characteristics, such as hydrophilicity and surface roughness^34^. Hydrophilicity is an important property for a bone-defect-filling material, as reported by Xu et al. when they synthesized a novel type of bone graft, BpNcCaP granules^35^. They found that BpNcCaP exhibited greater hydrophilicity and a higher propensity to be wetted by bodily fluids following implantation, potentially resulting in enhanced osteoconductivity compared to deproteinized bovine bone (Bio-Oss®). Besides, surface roughness plays an important role in the bioactivities of biomaterials^36^. We first characterized the surface hydrophilicity and roughness of the coated tantalum plates.

**Figure 3:**
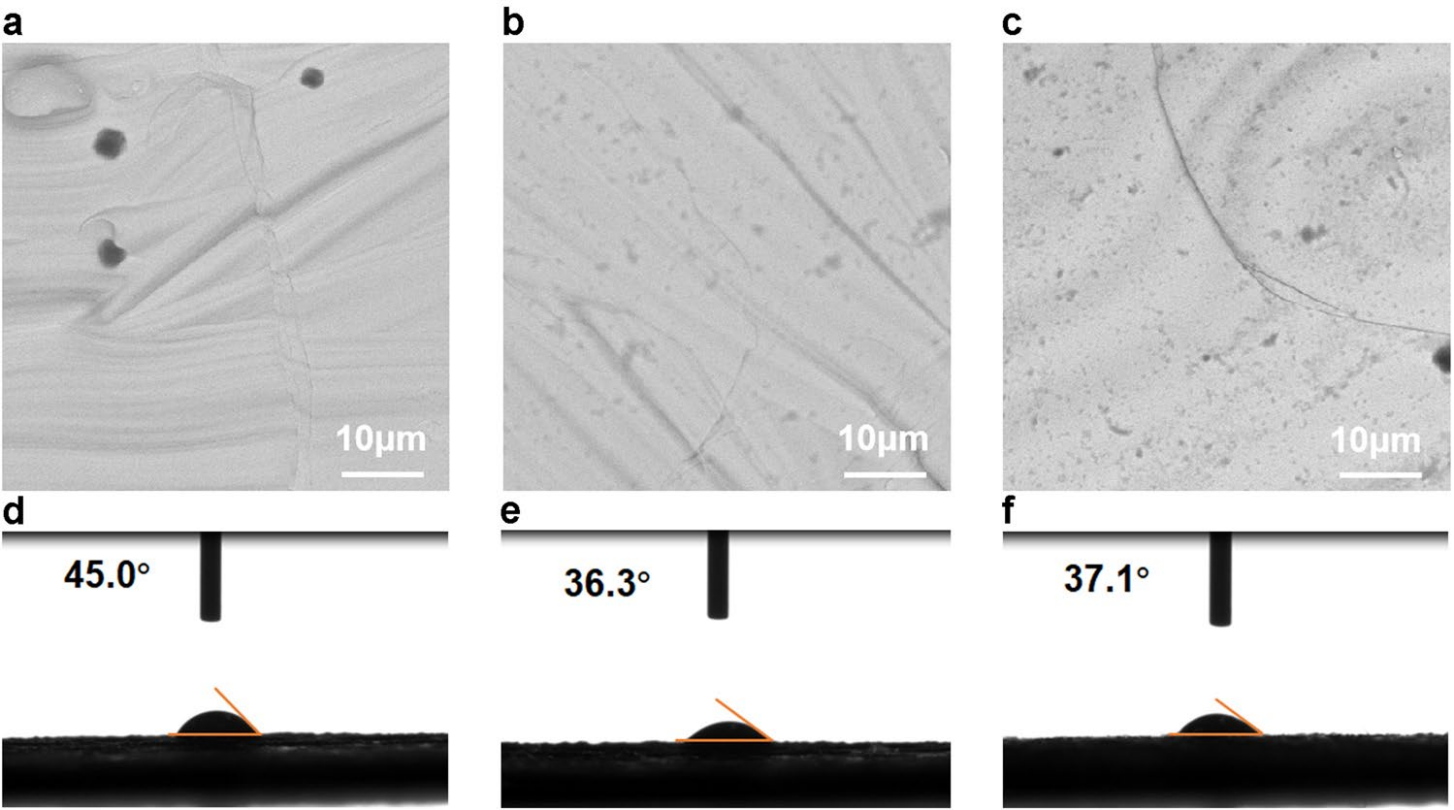
The results of the tests conducted on surface hydrophilicity and roughness. The surface roughness of blank tantalum (**a**), polyphenol-coated tantalum (**b**), and polyphenol-zinc-coated tantalum plates (**c**) were characterized under the scanning electron microscope. The average water contact angles of the blank tantalum (**d**), polyphenol-coated tantalum (**e**), and polyphenol-zinc-coated (**f**) tantalum plates were 45.0°, 36.3°, and 37.1°, respectively (n = 3).

Figure 3a–c shows the surface roughness of the blank tantalum, polyphenol-coated tantalum, and polyphenol-zinc-coated tantalum plates under SEM characterization. Figure 3d–f shows the water contact angles of the three groups of tantalum plates, which average were 45.0°, 36.3°, and 37.1° for the blank tantalum, polyphenol-coated tantalum, and polyphenol-zinc-coated tantalum plates, respectively (n = 3). In general, the smaller the contact angle of water on the material surface, the better its hydrophilicity^37,38^. Catechol groups are considered to be hydrophilic^39^. In this study, the surface of the tantalum plates became more hydrophilic owing to the introduction of a large number of catechol groups. Excellent surface hydrophilicity is also facilitating the application of biomaterials, making them more biocompatible^40^. The SEM analysis results showed that the surface of the tantalum plate was initially very smooth, but after the introduction of the polyphenol coating, a large number of aggregates could be seen on the surface of the plate, which was also reflected in the macroscopic color change. The results of hydrophilicity and roughness tests confirmed that the surface of the tantalum plate was coated with polyphenol and polyphenol zinc coatings. Studies have shown that the deposition of aggregates has the potential to enhance surface roughness^41,42^, and rough surfaces favor bone consolidation^43,44^. The rough surface of the implant promotes fibrin entrapment and mechanical stability in the host^45^. Moreover, rougher surfaces appear to be beneficial for adhesion, proliferation, and differentiation of human osteoblastic progenitor cells^46^. It is the principal function of osteoblasts to create new bone; therefore, osteoblast differentiation can promote bone formation^47^. Overall, rougher surfaces play an important role in bone repair and bone regeneration. In this study, surface modification increased the surface roughness of the tantalum plate, making it more suitable for the repair of mandibular and other defective surfaces.

### Cytocompatibility evaluation of the coated tantalum plates

**Figure 4:**
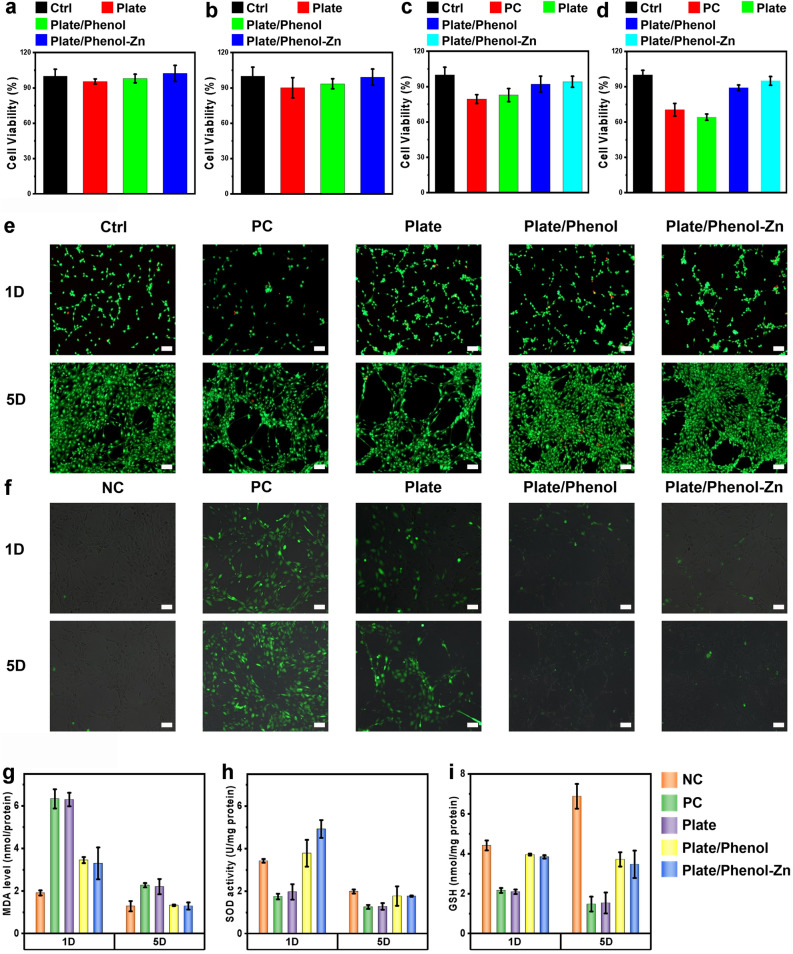
In vitro cell experiments. Ctrl group means cells cultured in the medium without any treatment, PC group means cells cultured in the medium after H_2_O_2_ treatment. (**a**) Cytotoxicity test results of materials after 1 day of culture. (**b**) Cytotoxicity test results of materials after 5 days of culture. (**c**) Protective effect of materials against oxidative damage induced by hydrogen peroxide after 1 day of culture. (**d**) Protective effect of materials against oxidative damage induced by hydrogen peroxide after 5 days of culture. (**e**) Results of staining analysis for live/dead cells after 1 day and 5 days of culture. The scale bar represents 10 µm. (**f**) Results of staining analysis for reactive oxygen species in cells after 1 day and 5 days of culture. The scale bar represents 10 µm. (**g**) Malondialdehyde assay results. (**h**) The results of superoxide dismutase activity determination. (**i**) Reduced glutathione assay results.

Relative cell viability was measured using an MTT assay. The experimental results are shown in Fig. 4a–d. The positive control group (PC) cells were affected by hydrogen peroxide, and the cell viability was lower than that of the normal control group (Ctrl). The blank tantalum plates (Plate) also showed similar results. However, cells in the polyphenol-coated (plate/phenol) and polyphenol-zinc-coated (plate/phenol-Zn) groups grew well, and the cell viability of the two groups was not significantly different from that of the control group, indicating that the surface-modified tantalum plate has good cytocompatibility and protection against hydrogen peroxide-induced cell damage. Live/dead staining was performed to confirm these results. Calcein-AM and propidium iodide were used to stain the live (green) and dead cells (red), respectively. The fluorescence images (Fig. 4e) showed that the polyphenol-coated and polyphenol-zinc-coated groups had higher cell density as well as proper cell morphology and proliferation than the PC group, further confirming that the excellent cytocompatibility of the surface-modified tantalum plates.

### *Evaluation of the antioxidative effects of the coated tantalum plates *in vitro

Usually, ROS produced during oxidative metabolism in organisms and eliminated by antioxidant defense systems maintain homeostasis^48,49^. Excessive ROS production and insufficient antioxidant responses can lead to oxidative stress^50,51^. The fluorescent probe DCFH-DA was used to measure the ROS generated within the cells. As shown in Fig. 4f, the ROS levels increased in cells treated with positive control and blank tantalum plates on the first day of culture. However, ROS levels in cells treated with coated tantalum plates were consistently lower than those in positive control cells, indicating lower ROS production in cells treated with coated tantalum plates. In addition, GSH and SOD are two important endogenous antioxidants that are crucial for maintaining the body's oxidative and antioxidant balance in vivo, as they scavenge ROS and protect cells from oxidative damage^52,53^. Lipid peroxidation can cause cell damage^54^, and MDA is one of the final products of lipid peroxidation. The level of MDA reflects the degree of oxidative damage in the body, and a significant increase in MDA level indicates that the cell membrane is severely oxidatively damaged^55,56^. We further determined the status of GSH/GSSG, SOD, and MDA in cells after treatment with H_2_O_2_ alone or in combination with coated tantalum plates for 1 day and 5 days. Compared to the normal control group, the levels of MDA (Fig. 4g) were significantly increased in cells treated with 20 mg/mL extract solution, while the levels of SOD (Fig. 4h) and GSH/GSSG (Fig. 4i) were significantly decreased. Compared with the positive control, the opposite results were obtained, indicating that the extract solution of coated tantalum plate may protect cells from damage caused by lipid peroxidation. There is evidence that biomaterials with antioxidant properties are more biocompatible than other materials^57^.

### Inflammatory response in the animal models

**Figure 5:**
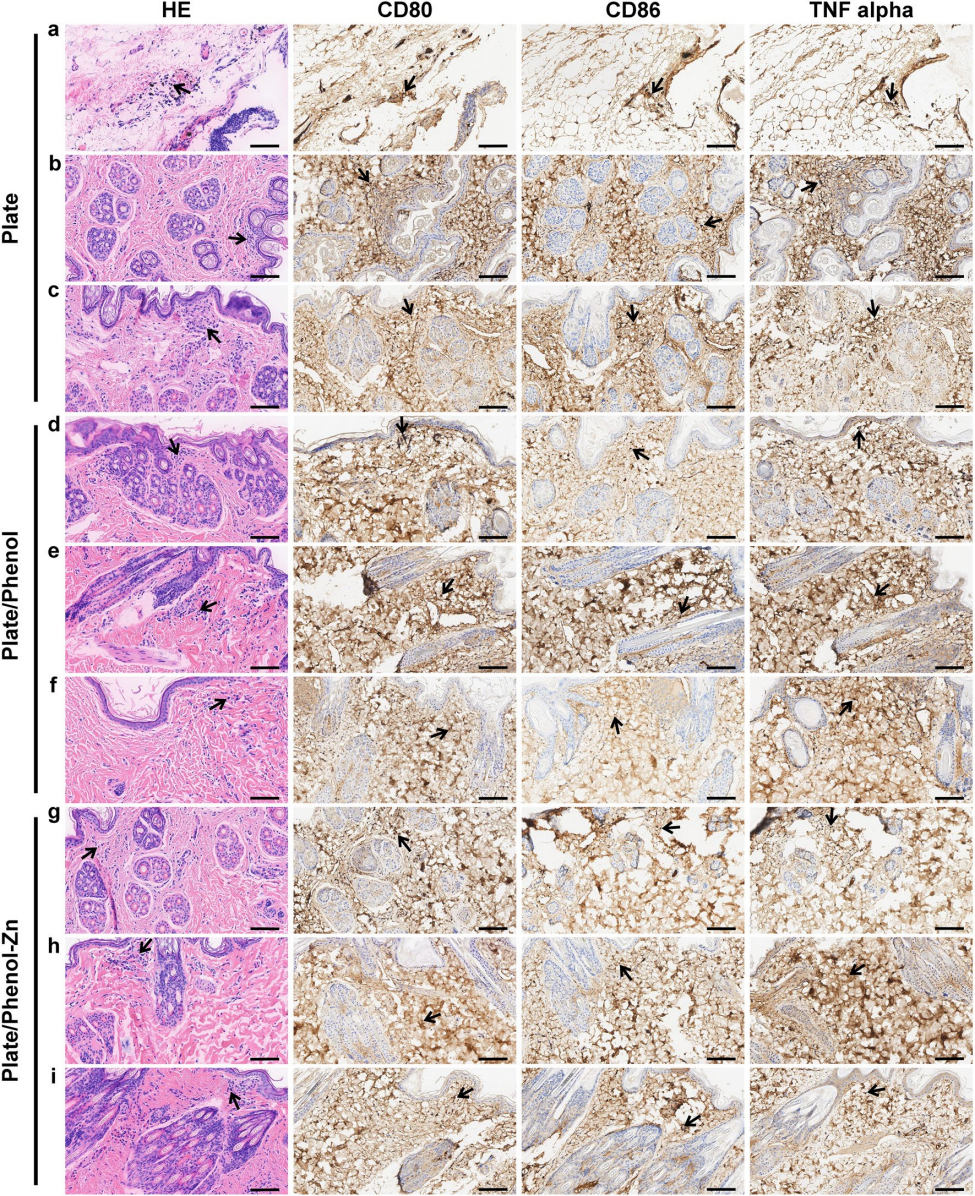
Hematoxylin and eosin (HE) staining (leftmost column) and immunohistochemistry staining (three rightmost columns) of implantation sites in rabbits. Arrows indicate inflammatory cells. Magnification: × 400, scale bars: 100 μ m. (**a**–**c**) HE and immunohistochemistry staining of three rabbits in the blank tantalum group. (**d**–**f**) HE and immunohistochemistry staining of three rabbits in the polyphenol-coated tantalum group. (**g**–**i**) HE and immunohistochemistry staining of three rabbits in the polyphenol-zinc-coated tantalum group.

Biomedical application of biomaterials demands good histocompatibility^58^. The histocompatibility of the implanted biomaterial can be assessed by the amount of infiltrated inflammatory cells^59^. To evaluate the histocompatibility of polyphenol-coated and polyphenol-zinc-coated tantalum plates, the inflammatory response was assessed by HE and immunohistochemistry staining. As shown in Fig. 5, HE staining demonstrated that the inflammatory response was slight in the rabbits of each group. A few inflammatory cells were observed in rabbits 1 (Fig. 5a) and 2 (Fig. 5b) of the blank tantalum plate group, while there were few inflammatory cells in rabbit 3 (Fig. 5c) of the blank tantalum plate group, and the rabbits in polyphenol-coated tantalum plate group (Fig. 5d–f) and the polyphenol-zinc-coated tantalum plate group (Fig. 5g–i). Additionally, immunohistochemistry analysis showed no more obvious positive expression of CD80, CD86, and TNF alpha in implantation sites of all groups (Fig. 5a–i). Moreover, Lu et al. reported an experimental study of implanting a novel porous tantalum implant in a rabbit anterior lumbar fusion model^60^. Histological results showed that no obvious local inflammatory response was found around or inside the tantalum implant, which is similar to our result. On the one hand, the above results suggested the low inflammatory stimulation and excellent histocompatibility of the tantalum implant. On the other hand, the inflammatory response is a highly complex and dynamic process^61^, it should be noted that only inflammatory response in the healing stage was analyzed in this study.

Our study involved a number of limitations. First, energy-dispersive X-ray spectrometer mapping was not performed in the present study. Second, the animals used in this study were all male rabbits and limited in number. Third, the assessment of inflammatory response was conducted at a single time point. Finally, our in vivo experiments involve a subcutaneous implantation model, rather than a craniomaxillofacial bone defect repair model. Therefore, more experiments are needed for further investigation.

## Conclusions

Using 3D printing and selective laser melting, we successfully fabricated tantalum plate models and modified the surface with polyphenol and polyphenol zinc coatings. The physicochemical properties of the coated tantalum plates were characterized, in vitro cell experiments were conducted using NIH-3T3 cells, and animal experiments were completed on New Zealand rabbits. Our experimental results showed that the surface-modified tantalum plate material has excellent cytocompatibility, antioxidant capabilities, and histocompatibility, which can protect cells from oxidative damage. Although further in vivo studies are needed to elucidate the effect of surface-modified tantalum plates on bone reactivity, these results suggest the potential of polyphenol-coated and polyphenol-zinc-coated tantalum plates as a matrix for craniomaxillofacial implant materials.

## Data Availability

The data presented in this study are included in the article. Further inquiries can be directed to the corresponding author.
